# The effectiveness of quality improvement collaboratives in improving stroke care and the facilitators and barriers to their implementation: a systematic review

**DOI:** 10.1186/s13012-021-01162-8

**Published:** 2021-11-03

**Authors:** Hayley J. Lowther, Joanna Harrison, James E. Hill, Nicola J. Gaskins, Kimberly C. Lazo, Andrew J. Clegg, Louise A. Connell, Hilary Garrett, Josephine M. E. Gibson, Catherine E. Lightbody, Caroline L. Watkins

**Affiliations:** 1 Applied Health Research hub (AHRh), University of Central Lancashire (UCLan), Preston, UK; 2 National Institute for Health Research Applied Research Collaboration North West Coast (NIHR ARC NWC), Liverpool, UK; 3 Faculty of Allied Health and Wellbeing, University of Central Lancashire (UCLan), Preston, UK; 4 Faculty of Health and Care, University of Central Lancashire (UCLan), Preston, UK

**Keywords:** Quality improvement collaborative, Stroke, Facilitators, Barriers, Effectiveness, Systematic review

## Abstract

**Background:**

To successfully reduce the negative impacts of stroke, high-quality health and care practices are needed across the entire stroke care pathway. These practices are not always shared across organisations. Quality improvement collaboratives (QICs) offer a unique opportunity for key stakeholders from different organisations to share, learn and ‘take home’ best practice examples, to support local improvement efforts. This systematic review assessed the effectiveness of QICs in improving stroke care and explored the facilitators and barriers to implementing this approach.

**Methods:**

Five electronic databases (MEDLINE, CINAHL, EMBASE, PsycINFO, and Cochrane Library) were searched up to June 2020, and reference lists of included studies and relevant reviews were screened. Studies conducted in an adult stroke care setting, which involved multi-professional stroke teams participating in a QIC, were included. Data was extracted by one reviewer and checked by a second. For overall effectiveness, a vote-counting method was used. Data regarding facilitators and barriers was extracted and mapped to the Consolidated Framework for Implementation Research (CFIR).

**Results:**

Twenty papers describing twelve QICs used in stroke care were included. QICs varied in their setting, part of the stroke care pathway, and their improvement focus. QIC participation was associated with improvements in clinical processes, but improvements in patient and other outcomes were limited. Key facilitators were inter- and intra-organisational networking, feedback mechanisms, leadership engagement, and access to best practice examples. Key barriers were structural changes during the QIC’s active period, lack of organisational support or prioritisation of QIC activities, and insufficient time and resources to participate in QIC activities. Patient and carer involvement, and health inequalities, were rarely considered.

**Conclusions:**

QICs are associated with improving clinical processes in stroke care; however, their short-term nature means uncertainty remains as to whether they benefit patient outcomes. Evidence around using a QIC to achieve system-level change in stroke is equivocal. QIC implementation can be influenced by individual and organisational level factors, and future efforts to improve stroke care using a QIC should be informed by the facilitators and barriers identified. Future research is needed to explore the sustainability of improvements when QIC support is withdrawn.

**Trial registration:**

Protocol registered on PROSPERO (CRD42020193966).

**Supplementary Information:**

The online version contains supplementary material available at 10.1186/s13012-021-01162-8.

Contributions to the literature

- This paper presents the first systematic review that has utilised the Consolidated Framework for Implementation Research (CFIR) to map facilitators and barriers to using a quality improvement collaborative (QIC) in improving stroke care.
- It highlights the effectiveness of QICs in improving clinical processes in stroke services and the importance of key factors that could be used to inform future efforts of planning and executing a QIC to successfully implement improvements in stroke care.
- This review identified a lack of patient and carer involvement, and consideration of health inequalities, in improving stroke care through the use of a QIC.

## Background

Stroke is one of the leading causes of death and disability worldwide [1]. Despite declines in age-standardised stroke incidence and mortality rates in recent years, the global burden of stroke remains high with over 80 million stroke survivors worldwide [1, 2]. To successfully reduce the negative impacts of stroke, high-quality health and care practices are needed across the entire stroke care pathway. Reorganising stroke services and implementing changes at a system-level are increasingly being recognised as ways of enhancing coordination across the pathway, optimising care processes, and improving outcomes for stroke patients [3–5]. Implementing these transformative changes in stroke care is likely to involve a critical mass of stakeholders across different organisations and will require the application of effective quality improvement (QI) methodologies.

Whilst there are many examples of good stroke care practices, these are not always shared between organisations. Quality improvement collaboratives (QICs) offer a unique opportunity for key stakeholders from different organisations to take part in a series of collaborative activities [6]. The QIC approach, first formalised by the Institute for Healthcare Improvement (IH), is a short-term structured programme, usually between 6 and 15 months, designed to support ‘breakthrough’ improvement in a focused topic area [7]. Teams from different organisations are brought together in ‘learning sessions’ to share and learn best practices and QI methods, and ‘take home’ learning to their organisation to test changes locally in ‘action periods’ [7]. Previous systematic reviews have evaluated the impact of QICs, reporting largely positive effects on improvement measures [6, 8]. Attempts to shed light on the potential determinants of QIC success have proposed the influence of external support [9], leadership [9], team functioning [9, 10], and collaborative learning [10, 11]. However, this literature has emphasised the need for further exploration of whether QIC effectiveness is dependent on the focus (e.g. clinical population), and if there are specific contextual factors that support or hinder QIC success [6, 8–10]. The importance of involving patients and carers in decisions about improving the care they receive [12], and the consideration of health inequalities when improving health and care services [13], is widely recognised, but to date, no review of QICs has examined the extent to which patients and carers were involved, or health inequalities were considered.

To build on previous QIC reviews, this systematic review assessed the effectiveness of QICs for driving improvements in stroke care and used the Consolidated Framework for Implementation Research (CFIR) [14] to explore the facilitators and barriers to using a QIC to improve care for this clinical population. The review also sought to consider the extent to which QICs in stroke care involved patients and carers and considered health inequalities.

## Methods

### Searches

This systematic review was registered with PROSPERO (CRD42020193966) and designed in accordance with recognised guidance and reporting standards (see Additional file 1 for the Preferred Reporting Items for Systematic Review and Meta-Analysis (PRISMA) checklist [15]). Studies were identified through searching five electronic databases (MEDLINE, CINAHL, EMBASE, PsycINFO, and Cochrane Library) from their inception to 5^th^ June 2020 and were limited to studies published in English. A search strategy using a combination of Medical Subject Headings and keywords related to ‘stroke’ and ‘quality improvement collaborative’ was developed with the assistance of an information specialist (see Additional file 2). Additional studies were identified through screening reference lists of included studies and relevant reviews.

### Study selection

Studies of any design referring to a QIC conducted in an adult stroke care setting, which reported primary effect measures and/or perspectives of participating multidisciplinary stroke teams, were included. The QIC approach was defined in line with previous reviews [6, 8, 9], consisting of the following core elements: (1) a specified topic; (2) clinical and QI experts working together; (3) multiple teams from multiple sites participating; (4) a model or framework for improvement with multiple tests of change; and (5) a series of structured collaborative activities in a given timeframe, involving learning sessions and visits from mentors and facilitators. Conference proceedings and reviews were excluded from the review. Two reviewers independently screened the titles and abstracts of all retrieved citations against the eligibility criteria using Rayyan [16]. Full texts of potentially relevant citations were then obtained and independently assessed by two reviewers. Disagreements at any stage were resolved through discussion with a third reviewer, and where necessary the wider review team. Reasons for exclusion at full-text screening were documented.

### Data extraction and quality assessment

Data was extracted from the included studies by one reviewer using a pre-piloted form in Microsoft Excel, and checked by a second for completeness and accuracy. Any disagreements were resolved through discussion with a third reviewer. The following data items were extracted from each study: authors, year of publication, country, aim, study design and setting, improvement area, QIC description and components, and any relevant outcomes. The extent to which patients and carers were involved, and health inequalities considered, was also noted. Data relating to the factors influencing stroke care improvement when using a QIC was extracted, in addition to those specifically labelled as facilitators and barriers. The Mixed Methods Appraisal Tool (MMAT), a critical appraisal tool designed for reviews which include quantitative, qualitative and mixed methods studies [17], was used to assess the methodological quality of included studies.

### Data synthesis

Detailed summaries of the study characteristics were collated. A vote-counting method based on the direction of effect was used to identify if there was any evidence of an effect in the included studies [18]. This approach was used due to heterogeneity observed in the studies, particularly in the outcomes assessed, and has been previously used in a similar review assessing the effectiveness of QI interventions [19]. For each outcome type (process, patient, and other), studies were categorised into five groups based on the ratio of outcomes demonstrating positive directional change, either from baseline to end of the study or when an intervention group was compared to a control group: (1) all outcomes; (2) more than half of the outcomes; (3) half of the outcomes; (4) less than half of the outcomes; and (5) no outcomes.

Extracted facilitators and barriers were mapped to the Consolidated Framework for Implementation Research (CFIR) [14] by one reviewer and verified by a second. The CFIR is comprised of five key domains (intervention characteristics, outer setting, inner setting, characteristics of individuals, and the implementation process), each containing constructs enabling the exploration of factors that influence implementation success [14]. This framework was selected as it focuses on organisational and contextual factors related to implementation, which was identified as most suitable for the collaborative nature of a QIC. It also served as a structure to explore factors across different study types. Thematic analysis was used to categorise facilitators and barriers for each relevant construct of the CFIR [20]. This stage was divided equally between two reviewers, with uncertainties resolved through discussion.

### Patient and public involvement in the review

A member of the public worked with researchers to develop the data extraction form, ensuring that the extent of patient and carer involvement, and whether improvements were patient-centred, were considered when extracting data, and reviewed this paper.

## Results

**Fig. 1:**
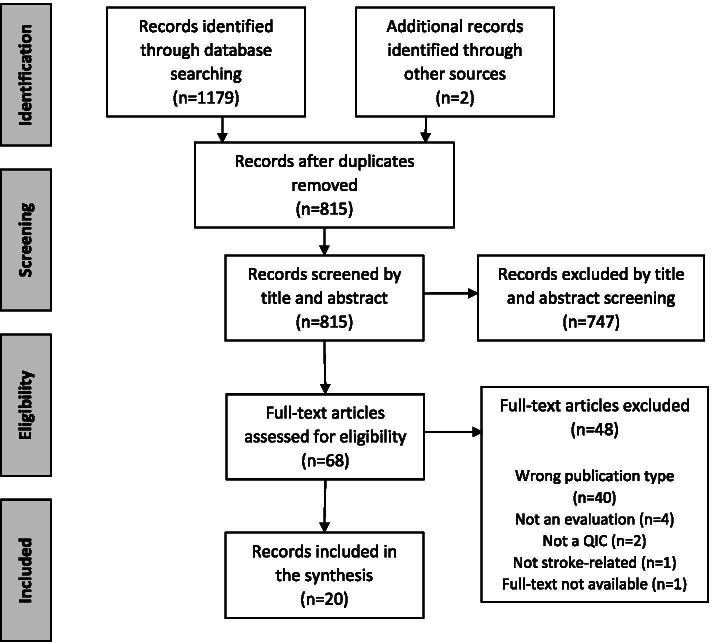
PRISMA flow diagram

The search strategy retrieved a total of 1179 citations. After the removal of duplicates, 815 citations were screened based on title and abstract, of which 68 records underwent full-text assessment. A total of 20 papers were identified for inclusion in the review, including two additional papers found through citation checking (Fig. 1).

### Study characteristics

Twenty papers describing 12 QICs used in stroke care were included; four randomised controlled trials [21–26], four cross-sectional studies [27–30], three interrupted time series studies [31–34], four before-and-after studies [35–38], and two qualitative studies [39, 40]. A summary of the included QICs is presented in Table 1. QICs were conducted in the USA [23, 29, 33–35, 38], UK [21, 31], Netherlands [22, 37], Australia [24] and Taiwan [36] between 2005 and 2020. Most QICs [21–24, 29, 31, 33–37] focused on improving urgent and/or acute stroke care. Key improvement areas included increasing thrombolysis treatment rates [22, 24, 29, 34, 36, 37], accurate and timely stroke screening and documentation [21, 23, 31, 33, 35–37], and increasing compliance in the full delivery of care bundles [21, 31]. Nine QICs took place in secondary care settings (e.g. hospitals) [21–24, 28, 29, 34–36], two QICs were based in pre-hospital care (e.g. emergency services) [31, 33], and one QIC was based in a primary care setting (e.g. general practice) [38]. One QIC took place across more than one setting type [28, 37], with stroke services from hospitals, rehabilitation organisations and nursing homes participating. The number of organisations participating in the QICs varied; some had between 10 to 15 sites [22, 23, 31, 34, 35], whilst others had between 20 and 24 sites [21, 24, 28, 33, 36]. Professionals involved in the QICs included QI experts, doctors, managers, nurses, and allied health professionals; some of whom were identified as specialist stroke clinicians and practitioners. There was variability in some QIC components; the number of learning sessions (from two to five), local QI methods used (plan-do-study-act cycles, driver diagrams, process maps), length of the QIC (from 6 to 48 months), and additional activities (teleconferences, workshops, site-based meetings). Most QICs used electronic/web-based data systems to measure performance [21–24, 33, 35, 37], and four QICs specified the use of a national registry [21, 35, 37, 38].

**Table 1:** Summary of included QICs

Name of QIC	Main QIC publication – author (year)	Country	Study design	Stroke care pathway stage	Main improvement areas/s	Number of study sites (intervention)	Duration of QIC (in months)	Outcome type (direction of effect)	Refs
Massachusetts EMS Stroke QIC	Daudelin et al. (2013)	USA	ITS	Urgent care	Prehospital stroke screening and documentation	17	36	Process (positive)	[33]
PRomoting ACute Thrombolysis in Ischemic StrokE (PRACTISE)	Dirks et al. (2011)	Netherlands	RCT	Urgent and acute care	Thrombolysis treatment rates	12 (6)	24	Process (positive) Patient (no effect) Other (positive)	[22, 25]
University of Best Practices (UBP) – “Be There San Diego”	Fulton et al. (2017)	USA	BA	Prevention	Reduce CVD morbidity and mortality	Unclear	36	Patient (no effect)	[38]
The Breakthrough Collaborative in Stroke	Hsieh et al. (2016)	Taiwan	BA	Urgent and acute care, secondary prevention	Various	24	12	Process (positive) Patient (no effect)	[36]
Thrombolysis Implementation in Stroke (TIPS)	Levi et al. (2020)	Australia	RCT	Urgent care	Thrombolysis treatment rates	20 (10)	16	Process (positive) Patient (no effect) Other (no effect)	[24, 26, 30]
Stroke Collaborative Reaching for Excellence (SCORE)	O’Neill et al. (2012)	USA	CS	Acute care	Various	56	48	Other (no effect)	[29]
Stroke 90:10	Power et al. (2014)	England	RCT	Acute and rehabilitation care	Delivery of early hours and rehabilitation care bundle	21 (10)	30	Process (positive) Other (no effect)	[21, 39]
QUality Enhancement for Speedy Thrombolysis in Stroke (QUESTS)	Prabhakaran et al. (2016)	USA	ITS	Urgent and acute care	Thrombolysis treatment times	15	12	Process (positive) Patient (positive)	[34]
Stroke Collaborative I and II	Schouten et al. (2008)	Netherlands	BA	Acute and rehabilitation care, long-term support	Length of hospital stay/discharge delay, and set up of integrated stroke services	23	16	Process (positive) Patient (positive)	[28, 37]
Ambulance Services Cardiovascular Quality Initiative (ASCQI)	Siriwardena et al. (2014)	England	ITS	Urgent care	Delivery of prehospital care bundle	12	25	Process (positive) Other (no effect)	[27, 31, 32]
Michigan Acute Stroke Care Overview and Treatment Surveillance System Quality Improvement Project (MASCOTS QIP)	Stoeckle Roberts et al. (2006)	USA	BA	Acute care and secondary prevention	Various	13	6	Process (positive)	[35]
Intervention for Stroke Improvement using Redesign Engineering (INSPIRE)	Williams et al. (2015)	USA	RCT	Acute care	Deep vein thrombolysis and dysphagia screening rates	11 (5)	12	Process (positive) Other (no effect)	[23, 40]

*BA* before-and-after study, *CS* cross-sectional study, *CVD* cardiovascular and/or cerebrovascular disease, *ITS* interrupted times series study, *QIC* quality improvement collaborative, *RCT* randomised controlled trial

### Quality assessment

The MMAT revealed that most papers were of medium to high quality [21–27, 29–32, 34, 36–40]. Two papers which scored as low quality [28, 35] either confirmed or added to the findings and so were included. Reliability of findings on quality assessment decisions is referred to in Tables 2 and 3.

**Table 2:** Facilitators identified in the QICs mapped to the CFIR domains and constructs

CFIR domain	CFIR construct	Facilitators	Refs	Reliability of findings based on MMAT
**Intervention characteristics**	Adaptability	QIC participation highlighted possibilities for using the approach for other aspects of stroke care and other clinical conditions.	[31, 34]	Medium
	Complexity	Processes of care within a geographical area or where a specific team in responsible may be more susceptible to improvement using a QIC.	[21, 23]	Medium
**Outer setting**	Patient needs and resources	Greater patient feedback may change staff perceptions of improvement being more than just a ‘tick-box exercise’.	[27]	High
	Cosmopolitanism	Collaborative action facilitates the exchange of ideas, best practice, and experience.	[28, 33, 36, 39]	Low-high
		Collaborative action fosters relationships between groups, improving cooperation and an emphasis on achieving results.	[28, 29, 33, 39]	Low-high
	External policy and incentives	External factors such as national level efforts during the QIC can influence the level of success achieved by using this approach.	[23, 26, 29, 38]	Medium-high
**Inner setting**	Structural characteristics	Stroke teams that function well may be associated with well organised stroke services and successful QI.	[28, 37]	Low-medium
		Teams composed of professionals and management may be more effective at implementing successful improvements and making decisions.	[28, 37]	Low-medium
	Networks and communications	Communication of the QIC to the organisation fosters support, provides networking opportunities, and enables change.	[27–29, 33, 35]	Low-high
	Culture	Longer serving members of staff may be more positive towards innovation.	[27]	High
	Implementation climate: Compatibility	Resolutions for solving issues related to implementation include assigning responsibility to a named individual, establishing accountability, and devising new workable processes.	[34, 39]	Low-high
		Positive baseline performance for acute stroke care may be associated with positive QI outcomes.	[23]	Medium
	Implementation climate: Relative priority	Identifying shared agenda and goals can unite QIC teams and help to find solutions.	[39, 40]	High
	Implementation climate: Organisational incentives and rewards	Motivation for change can be encouraged by organisation recognising activities undertaken by stroke teams.	[39]	High
	Implementation climate: Goals and feedback	Clinical feedback to staff is helpful for fostering successful QI.	[23, 30, 31, 33, 35, 39]	Low-high
		Positive feedback mechanisms include annotated control charts, provider prompts (checklists), storyboards and knowledge translation strategies.	[23, 30, 31, 33, 35, 39]	Low-high
		Focusing on essential topics and specifying aims if both necessary and helpful for achieving improvement results within a limited timeframe.	[28, 39]	Low-high
	Implementation climate: Learning climate	Learning sessions motivate change through opportunities to share and learn best practices and become familiar with QI tools.	[33, 39]	Medium-high
		Access to teaching from experts facilitates improvement.	[35, 36]	Low-medium
		Improving the content and accessibility of learning sessions may increase QIC participation.	[28–30]	Low-high
	Readiness for implementation: Leadership engagement	Involving and engaging senior leaders in the QIC and communicating progress to them is associated with improvement.	[27–29, 31, 35, 39]	Low-high
	Readiness for implementation: Available resources	Realistic time and resources for services should be provided for improvements to be achieved.	[31, 35, 40]	Low-high
		Recording staff time spent and resources used on improvement activities can be used to assess cost-effectiveness.	[25]	Medium
	Readiness for implementation: Access to knowledge	Access to useful information empowers teams to develop greater knowledge of best practice, patient care and QI methods and enables the appropriate induction of new staff.	[25, 28, 31, 33, 35, 40]	Low-high
		Stroke services with less knowledge and experience of QI may be more amenable to the approaches employed in a QIC.	[37]	Medium
**Individual characteristics**	Knowledge and beliefs about the intervention	Engagement with staff helps to foster a positive attitude towards changes implemented from the collaborative.	[27, 31]	Medium-high
	Self-efficacy	When staff understand the value of a QIC for improving patient care, it is a motivator for change.	[31, 39]	Medium-high
	Individual identification with organisation	The opportunity to work with other organisations and see what they are doing is a motivator for change.	[39]	High
	Other personal attributes	Individual or team characteristics have an impact on levels of enthusiasm and motivation.	[28]	High
**Process**	Engaging: Champions	Engaging and stimulating teams throughout the QIC is essential in encouraging improvements for patient care.	[27–29, 31, 39]	Low-high
		Interacting with leaders in meetings provides opportunities to discuss care and facilitates clinical engagement in QI activities.	[35]	High
	Engaging: external change agents	External facilitators empower teams to take ownership of the changes and provide support to clinicians on how best to navigate changes across services.	[40]	High
	Executing	Best practice examples were adopted by participating hospitals and may mediate improvements.	[34, 36]	Medium
		Consistency in employing the QIC approach and team participation, considering sustainability of changes, may support continued improvement.	[28, 29, 35]	Low-high
		A structured project approach, focusing on measurable outcomes, stimulates action and efficiency in stroke care.	[25, 28]	Low-medium
	Reflecting and evaluating	Monthly monitoring data encourages teams to reflect on their current practice, celebrate success and identify areas for improvement.	[39]	High

**Table 3:** Barriers identified in the QICs mapped to the CFIR domains and constructs

CFIR domain	CFIR construct	Barriers	Refs	Reliability of findings based on MMAT
**Intervention characteristics**	Complexity	QI processes are difficult to implement in a short period of time due to their associated complexities.	[28, 34, 35]	Low-medium
**Outer setting**	Patient needs and resources	QI in care may not be achievable in all stroke patients.	[26, 35, 36]	Low-medium
	Cosmopolitanism	Collaborative action can be undermined by: the effort required, lack of perceived benefit, negative comparisons, lack of contribution and resentment.	[28, 34, 39]	Low-high
	External policy and incentives	QIC participation can be hindered by not securing external support and having little to no experience of previous QI initiatives.	[34–36]	Low-medium
**Inner setting**	Structural characteristics	Organisational challenges such as staff turnover, changes to stroke service structure and available resources can have a negative impact of implementation, engagement, and motivation.	[22, 24, 28, 29, 31, 40]	Low-high
	Networks and communications	Collaboration over the phone may not be effective for providing support and meeting need.	[29, 40]	High
	Culture	QIC team members may perceive organisations as slow to change and lacking in innovative culture.	[27, 40]	High
	Implementation climate: Compatibility	Scheduling busy team members together for meetings is challenging.	[40]	High
	Implementation climate: Relative priority	Organisational priorities often take precedence above collaboration, innovation, and implementation.	[24, 27, 28, 33, 39]	Low-high
	Implementation climate: Organisational incentives and rewards	Lack of incentives for career learning and progression can create tension and affect morale.	[27, 39]	High
	Implementation climate: Goals and feedback	Lack of autonomy over improvement aims can affect the relevancy of changes and the degree of creativity a team can apply to them.	[28]	Low
	Implementation climate: Learning climate	Capacity and willingness to learn can impact the extent to which participants engage with the approaches employed in a QIC.	[29, 30, 39]	Medium-high
	Readiness for implementation: Leadership engagement	Unsupportive leadership can prevent teams from participating in the QIC and making improvements.	[28, 33, 39]	Low-high
	Readiness for implementation: Available resources	Insufficient staff time and resources allocated to QIC attendance and improvement activities, including data collection, significantly affects participation and success.	[24, 27, 28, 31, 33, 35, 37, 39, 40]	Low-high
	Readiness for implementation: Access to knowledge	Limited access to and experience with patient data tools and equipment is challenging.	[28, 35, 40]	Low-high
**Individual characteristics**	Knowledge and beliefs about the intervention	Perception of staff in different professions varies as to the need for intervention and the attitudes towards QICs.	[24, 27, 30, 35]	Low-high
	Other personal attributes	Motivation for change is susceptible to factors that are outside of the QICs control.	[31, 40]	Medium-high
**Process**	Engaging: Opinion leaders	Low actual levels or perceived levels of engagement with QI activities, particularly in clinicians, may impede improvement.	[24, 27, 31, 35]	Low-high
	Engaging: Champions	Local champions are not necessarily sufficient on their own to overcome some barriers and collaboration between local teams is required.	[24]	Medium
	Executing	Inconsistencies and delays in employing the QIC approach can have a negative impact on compliance, motivation, and improvement.	[22, 26, 31, 35]	Low-high
		When QIC support and resources are withdrawn, improvements may not be sustainable.	[23, 24, 26, 34, 35]	Low-medium

### Effectiveness of QICs in stroke care

Across the included studies, the effectiveness of QICs was categorised into three types of outcomes: process, patient, and other. Of the 14 studies (from ten QICs) with quantitative data, all reported process outcomes (e.g. door-to-needle times, blood glucose testing, discharge prescriptions) [21–26, 28, 31–37], seven studies (from six QICs) reported patient outcomes (e.g. mortality, quality of life, discharge delay) [22, 24, 28, 34, 36–38], and seven studies (from six QICs) reported other outcomes (e.g. staff engagement levels, perceptions of interventions, use of QI methods) [24, 25, 27, 29, 30, 39, 40]. All 14 studies reported a positive directional change in 50% to 100% of their process outcomes [21–26, 28, 31–37]; indicating that QICs were associated with improving clinical processes in stroke care. Of the seven studies reporting patient outcomes, three reported a positive directional change in 100% of these outcomes [28, 34, 37], two reported a positive directional change in less than half of their patient outcomes [22, 38], and two reported no change [24, 36]; suggesting that QICs may not be as effective in improving stroke patient outcomes. Of the seven studies reporting other outcomes, five reported no change [24, 27, 29, 39, 40], and two reported a positive change in these outcomes [25, 30]. Subgroup analyses, conducted by publication year, country, study setting, number of improvement areas, duration of QIC, number and length of learning sessions, and quality assessment judgement, identified no clear associations (see Additional file 3).

### Facilitators and barriers

Facilitators and barriers to implementing improvements in stroke care when using a QIC are summarised and mapped to the relevant CFIR domains and constructs in Tables 2 and 3, respectively. The following descriptions of the key facilitators and barriers identified are presented in the five CFIR domains.

#### Intervention characteristics

Six QICs reported factors related to the complexity and adaptability of the QIC intervention. Complex QI processes, or those requiring system re-design and multi-professional coordination, were more challenging, difficult to implement and unlikely to support change in the short-term [28, 34, 35]. Conversely, where indicators for change were kept simple and the stroke team had more control over them, improvement was more likely to be achieved [23, 39]. Identifying a specific geographical unit or designated team with recognised responsibility was viewed as important and may have encouraged a greater response to the QIC [23, 39]. Demonstrating the success of QI processes on delivery of care also highlighted their adaptability; for example, staff reported ‘spill over’ effects for other clinical conditions [31], and staff suggested that the QIC model could be applied to other aspects of stroke care like endovascular therapy [34].

#### Outer setting

Features of the external environment were identified as influencing improvement across all but one QIC [22]. External factors, such as the presence of national-level policies and incentives during the QIC [23, 26, 29, 38], or delays in securing contractual arrangements [35], influenced the extent to which organisations improved stroke care. Having little to no experience of previous QI initiatives, such as lack of familiarity with national data registries, meant improvement was less likely to happen for some organisations [34, 36]. The reported complexities associated with treating stroke, including challenging clinical presentations [36], being cared for in different areas of the hospital [35], and capturing accurate data on stroke onset [26], were barriers to achieving QI for all patients and all elements of stroke care.

Inter-organisational collaborative action, particularly during learning sessions, facilitated the exchange of ideas, best practices and experiences between organisations that would not normally work together [28, 33, 36, 39]. These exchanges stimulated teams to ‘take home’ learning to their organisation [28]. Relationships between organisations were fostered through the networking and communication opportunities offered by the QIC [28, 29, 33, 39]. It was reported that collaboration led to cooperation between teams, emphasis on the need for QI, and awareness of ‘being part of a chain of care’ [28]; and created ‘a sense of belonging’ and a ‘shared repertoire’ [39]. Though inter-organisational collaborative action was reported to facilitate improvement across some QICs [28, 29, 34, 36], the ‘Stroke 90:10’ QIC found that variability in performance, attendance, enthusiasm and contribution of teams created tension between organisations, which was not conducive to successful collaborative QI [39].

#### Inner setting

Factors in this domain were the most highly cited across all QICs. Insufficient organisational support (e.g. lack of prioritisation and inadequate allocation of time and resources for stroke QI) was reported as a significant barrier [24, 27, 28, 31, 33, 35, 37, 39, 40]. Structural changes (e.g. staff turnover) were also reported to negatively impact implementation [22, 24, 28, 29, 31, 40], and in one case led to an organisation withdrawing from the QIC [22]. QI was challenging for organisations that had limited access to equipment or patient data to measure performance [28, 35, 40]. Access to useful information delivered during QIC activities, however, empowered teams to develop knowledge of best practice, patient care and QI methods, which in turn facilitated stroke service improvement across some QICs [25, 28, 31, 33, 35, 40].

Leadership was noted to be associated with achieving improvement across some QICs [27–29, 31, 33, 35, 39]. Difficulties in obtaining support from leaders or changes in leadership hindered team participation in QI [28, 33, 39]. Some QICs highlighted how additional meetings and regular communication with leaders were successful tools to overcome these barriers and obtain buy-in from leaders to implement stroke care improvements [27–29, 31, 35]. Regular communication of QI activities and progress fostered support and recognition, provided intra-organisational networking opportunities and enabled change [28, 29, 33, 35, 39, 40]. Providing feedback to staff also supported improvement [23, 26, 31, 33, 35, 39]. Positive feedback mechanisms included audit and feedback [39], annotated control charts [31], provider prompts [31], and storyboards [35]. Learning sessions and access to experts motivated change by providing opportunities to share and learn best practices and become familiar with QI tools [33, 35, 36, 39]. Engagement with QI processes was influenced by capacity and willingness to learn [29, 30, 39] and tailoring the content and accessibility of learning sessions to suit participants [28–30, 40].

#### Characteristics of individuals

Individual characteristics were reported to influence improvement across six QICs. The perception of and response to QI processes differed depending on profession. Perceptions towards the effectiveness of thrombolysis were thought to have affected implementation for one QIC [24, 30], whilst another struggled to obtain support for QI measures due to a perception amongst emergency department staff that there were no quality issues surrounding stroke care [35]. Engaging staff from the outset may encourage more positive responses from colleagues towards the implementation of QI processes [27, 31]. Staff who perceived changes as a means of improving patient care, or creating a greater sense of purpose, were more likely to adopt them and look out to other organisations as well as their own [31, 39]. Other individual characteristics identified as influencing improvement included length of service [27], motivation [28, 31, 40], problem-solving [40], and enthusiasm [28].

#### Process

Ten QICs cited facilitators or barriers to QI associated with engaging appropriate individuals and executing the QIC intervention. Achieving improvement was difficult where there was low to moderate engagement in QI processes [24, 31], and where it was perceived that there was insufficient engagement from clinicians [27] or emergency department staff [35]. Engaging with all staff, particularly leaders, involved in delivering stroke care from the inception of the QIC and throughout was thought to facilitate change [27, 28, 31, 35, 39, 40]. Whilst external facilitators were found to empower teams to take ownership of changes in one QIC [40], another reported that sole reliance on local champions to support the change process was not necessarily sufficient and that more collaborative working was needed [24].

Inconsistencies in delivering the QIC intervention, for example implementation delays [31, 35], longer periods between learning sessions [22], and only having two learning sessions [30], negatively impacted motivation and improvement. Conversely, consistency in applying the QIC model with adequate team participation throughout and the use of a structured approach featuring measurable outcomes, supported improvement [25, 28, 29, 35]. Some QICs highlighted that whilst this intensive intervention facilitated initial improvement, when QIC support and resources were withdrawn, continued improvement might not be sustainable [23, 24, 34, 35]. QICs with longer-term data collection found no continued improvement in door-to-needle times [34], and declining thrombolysis rates [24], when the QIC ended.

### Patient and carer involvement and health inequalities

Patient and carer involvement rarely featured in the QICs. None undertook qualitative data collection of patient or carer perspectives of QI, or explored whether their experience had changed as a result of the QIC. An English ambulance service QIC concluded that as patients were the care receivers, their experiences should inform QI [27]. All but one QIC [38] were focused on improving clinical quality rather than patient-centred improvement areas, and only half of the QICs measured patient outcomes [22, 24, 34, 36–38]. Whilst unwarranted variation between stroke services was a motivation for improvement in two QICs [21, 28], the context of socioeconomic health inequalities associated with stroke was not present in most QICs. One USA QIC factored health insurance and poverty level into their analysis to assess whether QI activities decreased hospitalisations for stroke in all populations [38].

## Discussion

This systematic review assessed the effectiveness of QICs in improving stroke care and explored the facilitators and barriers associated with using the QIC approach. It was considered important given the possible benefits from using a QIC in reorganising stroke services and implementing system-level changes in stroke care. In line with previous QIC reviews [6, 8], the present review found that QICs support positive change for some outcome measures, particularly those related to improving clinical processes. Echoing concerns from these reviews [6, 8], evidence of effectiveness was limited due to the low methodological quality of some studies and the heterogeneity of study design, meaning that meta-analysis was not possible. Whilst QICs were associated with improving clinical processes in stroke care and to some extent patient outcomes, effects on staff engagement, perceptions, and uptake of QI methods were limited. The short-term and intensive nature of a QIC may have restricted the extent to which some measures could be affected. Patient-based outcomes or those related to individual behaviour or organisational change may require longer-term monitoring and embedding of QI processes. Few QICs assessed whether improvements continued or were sustained when the QIC ended. In those that had longer-term follow-up, outcomes had remained the same [34], or worsened [24]; suggesting that when QIC support was withdrawn, continued or even sustained, improvement may not be possible. It has been noted that encouraging a project-like approach to QI can be harmful for continuous improvement [41], supporting the idea that when a QIC ends, the gains achieved during the programme may attenuate as teams re-focus efforts on other aspects of care delivery.

Many factors identified by this review as supportive to QI were consistent with findings from other QIC reviews [9, 10], indicating they are not unique to this clinical population. Use of the CFIR domains to map facilitators and barriers has highlighted the importance of the inner and outer setting when using a QIC to improve stroke care. This substantiates results from the wider QI literature [42, 43], indicating that contextual factors within the organisation and external environment influence the extent to which improvement can be achieved. The positive effect of collaborative interaction (e.g. inter- and intra-organisational networking opportunities) identified, is also evident in previous explorations of QICs [10, 11], including in a recent realist review proposing collaborative ‘capacity building’ as a mechanism for change [9]. The present review’s findings, particularly those related to the influence of networking and access to information, corroborate several conclusions reached by Zamboni and colleagues [9]. Importantly, identifying engagement as a key facilitator further supports the present view that engagement plays a vital role in harnessing QI within an organisation [9, 43]. Despite this emphasis, greater efforts to understand how to increase engagement, who to engage with, and at what stages in the process, could better inform how to optimise a QIC in stroke care.

Given the prominence of factors within the inner setting, QIC success may rely on an organisation’s capacity to participate. This may form the basis of key criteria to be met before subscribing to the approach. Addressing barriers associated with a lack of organisational support, consistently identified across the wider QI literature [41–43], is likely to support stroke care QI. Alternative QIC formats such as virtual collaborative events may alleviate some barriers associated with QIC participation (e.g. time commitment) [44]. Intervention and individual characteristics specific to stroke were identified as barriers to implementing improvements using a QIC. In addition to patient-level barriers, such as challenging clinical presentations and the accuracy of stroke data, complex changes in stroke which involved different hospital areas and teams were more difficult to achieve with a QIC. The focus for future QICs may therefore be limited to implementing smaller process changes in stroke care and only with certain cohorts of stroke patients. The perceptions of and response to QI, and in some cases the intervention itself (e.g. thrombolysis), differed depending on profession across some QICs. Given that QICs were less likely to be associated with increasing engagement, changing perceptions, or increasing the uptake of QI methods; exploring ways in which to address these aspects of QI in stroke care deserves attention in future studies.

Patients and carers were not involved in the QICs, and the context of health inequalities was rarely considered. Despite the importance of involving care receivers in improving health services [3, 12], evidence of how and in what circumstances to involve them in QI, remains limited. The lack of consideration of health inequalities in the QICs was unsurprising, as those conducted in secondary care settings tend to focus on administering treatments for presenting health conditions rather than on addressing the underlying determinants of health and equitable access to services.

The findings from this review could be used to inform practice and the direction of future research. First, factors found to influence improvement, such as engagement and organisational support, should be considered by those planning future QIC initiatives in stroke care to enhance chances of success. Developing a tool to assess the presence or absence of the factors found in this review could be useful to support a healthcare organisation in the effective implementation of a QIC to improve stroke care. Second, the lack of stroke patient and carer involvement identified in this review suggests that there is a need for future studies to explore the ways in which patients and carers could be involved in a QIC. Utilising qualitative methodology similar to other participatory projects in QI [45, 46], to characterise how patient and carer experience and knowledge can contribute to a QIC may help to evaluate if their involvement could support a more patient-centred approach to implementing improvements in stroke services. As the focus of many QICs was implementing smaller process changes in discrete parts of the stroke care pathway, future research should be conducted to identify how system-level change can be achieved and whether a QIC would support this. Such studies could adopt the conceptual framework for implementing major system change developed by Fulop and colleagues [47], employing a QIC as the implementation approach and evaluate its potential to influence outcomes associated with system-level change. Lastly, there is a need for further exploration of the sustainability of improvements once QIC support is withdrawn, and how to support continued improvement and ongoing inter-organisational networking. Applying theories as identified in a recent systematic review [48], could identify potential avenues for sustainment strategies and advance understanding of how to sustain improvement and networking when a QIC ends.

This systematic review was conducted using standardised methods, a well-established implementation framework to consider facilitators and barriers, and included public involvement. In addition to searching five academic databases, scoping searches of the grey literature were conducted, and no additional records were identified. Though the searches were comprehensive, it is possible that some relevant papers may have been missed by not systematically reviewing those not published in English. QICs included in this review did not report negative changes across outcome measures, indicating a potential publication bias as QICs with negative findings are less likely to be published than those with positive results. In addition, the majority of studies reported process outcomes and very few reported patient outcomes, and therefore whilst QICs appear to be associated with improving clinical processes in stroke, it should not be assumed that these are directly associated with patient improvements and could highlight a potential shortfall of research in this area [49].

## Conclusion

QICs are associated with improving clinical processes in stroke care; however, their short-term nature means uncertainty remains as to whether they benefit patient outcomes. Although helpful with improving elements of the stroke care pathway, evidence around using QICs to achieve system-level change is equivocal. Further research is needed to explore the sustainability of improvements when QIC support is withdrawn. QIC implementation can be compromised by both individual and organisational level barriers. It is evident that engagement, communication, and access to best practice examples could be key to enhancing QIC success in improving stroke care. As a result, future efforts to drive stroke care improvement using a QIC should be informed by these facilitators and barriers.

## Supplementary Information

**Additional file 1.** PRISMA 2009 Checklist.

**Additional file 2.** Search terms.

**Additional file 3.** Effectiveness of QICs.

## Data Availability

All data generated or analysed during this study are included in this paper and its supplementary information files.

## Abbreviations

BA: Before-and-after study
CFIR: Consolidated Framework for Implementation Research
CS: Cross-sectional study
CVD: Cardiovascular and/or cerebrovascular disease
IHI: Institute for Healthcare Improvement
ITS: Interrupted time series study
MMAT: Mixed Methods Appraisal Tool
PRISMA: Preferred Reporting Items for Systematic Reviews and Meta-Analyses
QI: Quality improvement
QIC: Quality improvement collaborative
RCT: Randomised controlled trial
UK: United Kingdom
USA: United States of America
