# Supplementary material for: The effectiveness of quality improvement collaboratives in improving stroke care and the facilitators and barriers to their implementation: a systematic review
Source: Implement Sci. 2021 Nov 3;16:95. doi: 10.1186/s13012-021-01162-8 (PMC8564999; doi:10.1186/s13012-021-01162-8)
Supplement: Supplementary file 2 — Additional file 2. Search terms. [file 13012_2021_1162_MOESM2_ESM.docx]

Additional file 2. Search terms

----------------------------------------------------------------------

1. cerebrovascular disorders/ or exp basal ganglia/ or cerebrovascular disease/ or exp brain ischemia/ or exp carotid artery diseases/ or exp intracranial arterial diseases/ or exp intracranial arteriovenous malformations/ or exp "intracranial embolism and thrombosis"/ or exp intracranial hemorrhages/ or stroke/ or exp brain infarction/

2. brain injuries/ or brain injury, chronic/

3. (stroke$ or cva or poststroke or post-stroke or cerebrovasc$ or cerebral vascular).tw

4. ((cerebral or cerebellar or brain$ or vertebrobasilar) adj5 (infarct$ or isc?emi$ or thrombo$ or emboli$ or apoplexy)).tw

5. ((cerebral or brain or subarachnoid) adj5 (haemorrhage or hemorrhage or haematoma or hematoma or bleed$)).tw

6. 1 or 2 or 3 or 4 or 5

7. ((learning or improvement or quality or breakthrough) adj3 collaborat*).mp

8. (Breakthrough adj (series or approach* or network* or project*)).mp

9. collaborative network*.mp

10. improvement network*.mp

11. (quality improv* adj5 collaborat*).mp

12. (model* adj2 improvement).mp

13. or/7-12

14. 6 and 13

-----------------------------------------------------------------------------
