# Supplementary material for: The effectiveness of quality improvement collaboratives in improving stroke care and the facilitators and barriers to their implementation: a systematic review
Source: Implement Sci. 2021 Nov 3;16:95. doi: 10.1186/s13012-021-01162-8 (PMC8564999; doi:10.1186/s13012-021-01162-8)
Supplement: Supplementary file 3 — Additional file 3. Effectiveness of QICs. [file 13012_2021_1162_MOESM3_ESM.docx]

Additional file 3. Effectiveness of QICs

Table A1. QIC studies evaluating change in process outcomes

|  |  | **Number and % of studies reporting a positive directional change for process outcomes** | | | | |
| --- | --- | --- | --- | --- | --- | --- |
|  | **Number of studies** | **All outcomes** | **Over half outcomes** | **Half outcomes** | **Under half outcomes** | **No outcomes** |
| All studies | 14 | 5 (36%) | 8 (57%) | 1 (7%) | 0 (0%) | 0 (0%) |
| **Publication year** | | | | | | |
| 2001-2005 | 1 | 0 (0%) | 1 (100%) | 0 (0%) | 0 (0%) | 0 (0%) |
| 2006-2010 | 2 | 0 (0%) | 2 (100%) | 0 (0%) | 0 (0%) | 0 (0%) |
| 2011-2015 | 6 | 4 (67%) | 2 (33%) | 0 (0%) | 0 (0%) | 0 (0%) |
| 2016-2020 | 5 | 1 (20%) | 3 (60%) | 1 (20%) | 0 (0%) | 0 (0%) |
| **Country** | | | | | | |
| Australia | 2 | 1 (50%) | 0 (0%) | 1 (50%) | 0 (0%) | 0 (0%) |
| England | 3 | 2 (67%) | 1 (33%) | 0 (0%) | 0 (0%) | 0 (0%) |
| Netherlands | 4 | 1 (25%) | 3 (75%) | 0 (0%) | 0 (0%) | 0 (0%) |
| Taiwan | 1 | 0 (0%) | 1 (100%) | 0 (0%) | 0 (0%) | 0 (0%) |
| USA | 4 | 1 (25%) | 3 (75%) | 0 (0%) | 0 (0%) | 0 (0%) |
| **Study setting** | | | | | | |
| Primary care | 0 | 0 (0%) | 0 (0%) | 0 (0%) | 0 (0%) | 0 (0%) |
| Pre-hospital care | 3 | 3 (100%) | 0 (0%) | 0 (0%) | 0 (0%) | 0 (0%) |
| Secondary care | 11 | 2 (18%) | 8 (73%) | 1 (9%) | 0 (0%) | 0 (0%) |
| Tertiary care | 0 | 0 (0%) | 0 (0%) | 0 (0%) | 0 (0%) | 0 (0%) |
| Social care | 2 | 0 (0%) | 2 (100%) | 0 (0%) | 0 (0%) | 0 (0%) |
| **Number of improvement areas** | | | | | | |
| 1 | 5 | 2 (40%) | 2 (40%) | 1 (20%) | 0 (0%) | 0 (0%) |
| 2 | 2 | 0 (0%) | 2 (100%) | 0 (0%) | 0 (0%) | 0 (0%) |
| 3 | 2 | 2 (100%) | 0 (0%) | 0 (0%) | 0 (0%) | 0 (0%) |
| 4 | 0 | 0 (0%) | 0 (0%) | 0 (0%) | 0 (0%) | 0 (0%) |
| 5+ | 5 | 1 (20%) | 4 (80%) | 0 (0%) | 0 (0%) | 0 (0%) |
| **Duration of QIC** | | | | | | |
| 6-11 months | 1 | 0 (0%) | 1 (100%) | 0 (0%) | 0 (0%) | 0 (0%) |
| 12-17 months | 7 | 1 (14%) | 5 (71%) | 1 (14%) | 0 (0%) | 0 (0%) |
| 18-23 months | 0 | 0 (0%) | 0 (0%) | 0 (0%) | 0 (0%) | 0 (0%) |
| 24-29 months | 3 | 2 (67%) | 1 (33%) | 0 (0%) | 0 (0%) | 0 (0%) |
| 30-35 months | 1 | 0 (0%) | 1 (100%) | 0 (0%) | 0 (0%) | 0 (0%) |
| 36+ months | 1 | 0 (0%) | 1 (100%) | 0 (0%) | 0 (0%) | 0 (0%) |
| Not reported | 1 | 1 (100%) | 0 (0%) | 0 (0%) | 0 (0%) | 0 (0%) |
| **Number of learning sessions** | | | | | | |
| 2 | 3 | 1 (33%) | 1 (33%) | 1 (33%) | 0 (0%) | 0 (0%) |
| 3 | 5 | 2 (40%) | 3 (60%) | 0 (0%) | 0 (0%) | 0 (0%) |
| 4 | 2 | 0 (0%) | 2 (100%) | 0 (0%) | 0 (0%) | 0 (0%) |
| 5 | 3 | 1 (33%) | 2 (67%) | 0 (0%) | 0 (0%) | 0 (0%) |
| Not reported | 1 | 1 (100%) | 0 (0%) | 0 (0%) | 0 (0%) | 0 (0%) |
| **Length of learning sessions** | | | | | | |
| 1/2 day | 1 | 0 (0%) | 1 (100%) | 0 (0%) | 0 (0%) | 0 (0%) |
| 1 day | 2 | 0 (0%) | 1 (50%) | 1 (50%) | 0 (0%) | 0 (0%) |
| 1-2 days | 1 | 0 (0%) | 1 (100%) | 0 (0%) | 0 (0%) | 0 (0%) |
| Not reported | 9 | 5 (56%) | 4 (44%) | 0 (0%) | 0 (0%) | 0 (0%) |
| **Quality assessment** | | | | | | |
| low | 2 | 0 (0%) | 2 (100%) | 0 (0%) | 0 (0%) | 0 (0%) |
| medium | 10 | 4 (40%) | 5 (50%) | 1 (10%) | 0 (0%) | 0 (0%) |
| high | 2 | 1 (50%) | 1 (50%) | 0 (0%) | 0 (0%) | 0 (0%) |

Table A2. QIC studies evaluating change in patient outcomes

|  |  | **Number and % of studies reporting a positive directional change for patient outcomes** | | | | |
| --- | --- | --- | --- | --- | --- | --- |
|  | **Number of studies** | **All outcomes** | **Over half outcomes** | **Half outcomes** | **Under half outcomes** | **No outcomes** |
| All studies | 7 | 3 (43%) | 0 (0%) | 0 (0%) | 2 (29%) | 2 (29%) |
| **Publication year** | | | | | | |
| 2001-2005 | 1 | 1 (100%) | 0 (0%) | 0 (0%) | 0 (0%) | 0 (0%) |
| 2006-2010 | 1 | 1 (100%) | 0 (0%) | 0 (0%) | 0 (0%) | 0 (0%) |
| 2011-2015 | 1 | 0 (0%) | 0 (0%) | 0 (0%) | 1 (100%) | 0 (0%) |
| 2016-2020 | 4 | 1 (25%) | 0 (0%) | 0 (0%) | 1 (25%) | 2 (50%) |
| **Country** | | | | | | |
| Australia | 1 | 0 (0%) | 0 (0%) | 0 (0%) | 0 (0%) | 1 (100%) |
| England | 0 | 0 (0%) | 0 (0%) | 0 (0%) | 0 (0%) | 0 (0%) |
| Netherlands | 3 | 2 (67%) | 0 (0%) | 0 (0%) | 1 (33%) | 0 (0%) |
| Taiwan | 1 | 0 (0%) | 0 (0%) | 0 (0%) | 0 (0%) | 1 (100%) |
| USA | 2 | 1 (50%) | 0 (0%) | 0 (0%) | 1 (50%) | 0 (0%) |
| **Study setting** | | | | | | |
| Primary care | 1 | 0 (0%) | 0 (0%) | 0 (0%) | 1 (100%) | 0 (0%) |
| Pre-hospital care | 0 | 0 (0%) | 0 (0%) | 0 (0%) | 0 (0%) | 0 (0%) |
| Secondary care | 6 | 3 (50%) | 0 (0%) | 0 (0%) | 1 (17%) | 2 (33%) |
| Tertiary care | 0 | 0 (0%) | 0 (0%) | 0 (0%) | 0 (0%) | 0 (0%) |
| Social care | 2 | 2 (100%) | 0 (0%) | 0 (0%) | 0 (0%) | 0 (0%) |
| **Number of improvement areas** | | | | | | |
| 1 | 4 | 2 (50%) | 0 (0%) | 0 (0%) | 1 (25%) | 1 (25%) |
| 2 | 1 | 1 (100%) | 0 (0%) | 0 (0%) | 0 (0%) | 0 (0%) |
| 3 | 0 | 0 (0%) | 0 (0%) | 0 (0%) | 0 (0%) | 0 (0%) |
| 4 | 0 | 0 (0%) | 0 (0%) | 0 (0%) | 0 (0%) | 0 (0%) |
| 5+ | 2 | 1 (50%) | 0 (0%) | 0 (0%) | 0 (0%) | 1 (50%) |
| Not reported | 1 | 0 (0%) | 0 (0%) | 0 (0%) | 1 (100%) | 0 (0%) |
| **Duration of QIC** | | | | | | |
| 6-11 months | 0 | 0 (0%) | 0 (0%) | 0 (0%) | 0 (0%) | 0 (0%) |
| 12-17 months | 5 | 3 (60%) | 0 (0%) | 0 (0%) | 0 (0%) | 2 (40%) |
| 18-23 months | 0 | 0 (0%) | 0 (0%) | 0 (0%) | 0 (0%) | 0 (0%) |
| 24-29 months | 1 | 0 (0%) | 0 (0%) | 0 (0%) | 1 (100%) | 0 (0%) |
| 30-35 months | 0 | 0 (0%) | 0 (0%) | 0 (0%) | 0 (0%) | 0 (0%) |
| 36+ months | 1 | 0 (0%) | 0 (0%) | 0 (0%) | 1 (100%) | 0 (0%) |
| Not reported | 0 | 0 (0%) | 0 (0%) | 0 (0%) | 0 (0%) | 0 (0%) |
| **Number of learning sessions** | | | | | | |
| 2 | 1 | 0 (0%) | 0 (0%) | 0 (0%) | 0 (0%) | 1 (100%) |
| 3 | 1 | 0 (0%) | 0 (0%) | 0 (0%) | 0 (0%) | 1 (100%) |
| 4 | 2 | 2 (100%) | 0 (0%) | 0 (0%) | 0 (0%) | 0 (0%) |
| 5 | 2 | 1 (50%) | 0 (0%) | 0 (0%) | 1 (50%) | 0 (0%) |
| Not reported | 1 | 0 (0%) | 0 (0%) | 0 (0%) | 1 (100%) | 0 (0%) |
| **Length of learning sessions** | | | | | | |
| 1/2 day | 1 | 0 (0%) | 0 (0%) | 0 (0%) | 1 (100%) | 0 (0%) |
| 1 day | 0 | 0 (0%) | 0 (0%) | 0 (0%) | 0 (0%) | 0 (0%) |
| 1-2 days | 0 | 0 (0%) | 0 (0%) | 0 (0%) | 0 (0%) | 0 (0%) |
| Not reported | 6 | 3 (50%) | 0 (0%) | 0 (0%) | 1 (17%) | 2 (33%) |
| **Quality assessment** | | | | | | |
| low | 1 | 1 (100%) | 0 (0%) | 0 (0%) | 0 (0%) | 0 (0%) |
| medium | 5 | 2 (40%) | 0 (0%) | 0 (0%) | 1 (20%) | 2 (40%) |
| high | 1 | 0 (0%) | 0 (0%) | 0 (0%) | 1 (100%) | 0 (0%) |

Table A3. QIC studies evaluating change in other outcomes

|  |  | **Number and % of studies reporting a positive directional change for other outcomes** | | | | |
| --- | --- | --- | --- | --- | --- | --- |
|  | **Number of studies** | **All outcomes** | **Over half outcomes** | **Half outcomes** | **Under half outcomes** | **No outcomes** |
| All studies | 7 | 1 (14%) | 0 (0%) | 0 (0%) | 1 (14%) | 5 (71%) |
| **Publication year** | | | | | | |
| 2001-2005 | 0 | 0 (0%) | 0 (0%) | 0 (0%) | 0 (0%) | 0 (0%) |
| 2006-2010 | 0 | 0 (0%) | 0 (0%) | 0 (0%) | 0 (0%) | 0 (0%) |
| 2011-2015 | 4 | 1 (25%) | 0 (0%) | 0 (0%) | 0 (0%) | 3 (75%) |
| 2016-2020 | 3 | 0 (0%) | 0 (0%) | 0 (0%) | 1 (33%) | 2 (67%) |
| **Country** | | | | | | |
| Australia | 2 | 0 (0%) | 0 (0%) | 0 (0%) | 1 (50%) | 1 (50%) |
| England | 2 | 0 (0%) | 0 (0%) | 0 (0%) | 0 (0%) | 2 (100%) |
| Netherlands | 1 | 1 (100%) | 0 (0%) | 0 (0%) | 0 (0%) | 0 (0%) |
| Taiwan | 0 | 0 (0%) | 0 (0%) | 0 (0%) | 0 (0%) | 0 (0%) |
| USA | 2 | 0 (0%) | 0 (0%) | 0 (0%) | 0 (0%) | 2 (100%) |
| **Study setting** | | | | | | |
| Primary care | 0 | 0 (0%) | 0 (0%) | 0 (0%) | 0 (0%) | 0 (0%) |
| Pre-hospital care | 1 | 0 (0%) | 0 (0%) | 0 (0%) | 0 (0%) | 1 (100%) |
| Secondary care | 6 | 1 (17%) | 0 (0%) | 0 (0%) | 1 (17%) | 4 (67%) |
| Tertiary care | 0 | 0 (0%) | 0 (0%) | 0 (0%) | 0 (0%) | 0 (0%) |
| Social care | 0 | 0 (0%) | 0 (0%) | 0 (0%) | 0 (0%) | 0 (0%) |
| **Number of improvement areas** | | | | | | |
| 1 | 3 | 0 (0%) | 0 (0%) | 1 (33%) | 1 (33%) | 1 (33%) |
| 2 | 1 | 0 (0%) | 0 (0%) | 0 (0%) | 0 (0%) | 1 (100%) |
| 3 | 1 | 0 (0%) | 0 (0%) | 0 (0%) | 0 (0%) | 1 (100%) |
| 4 | 0 | 0 (0%) | 0 (0%) | 0 (0%) | 0 (0%) | 0 (0%) |
| 5+ | 1 | 0 (0%) | 0 (0%) | 0 (0%) | 0 (0%) | 1 (100%) |
| Not reported | 1 | 0 (0%) | 0 (0%) | 0 (0%) | 0 (0%) | 1 (100%) |
| **Duration of QIC** | | | | | | |
| 6-11 months | 0 | 0 (0%) | 0 (0%) | 0 (0%) | 0 (0%) | 0 (0%) |
| 12-17 months | 3 | 0 (0%) | 0 (0%) | 0 (0%) | 1 (33%) | 2 (67%) |
| 18-23 months | 0 | 0 (0%) | 0 (0%) | 0 (0%) | 0 (0%) | 0 (0%) |
| 24-29 months | 1 | 0 (0%) | 0 (0%) | 0 (0%) | 0 (0%) | 1 (100%) |
| 30-35 months | 0 | 0 (0%) | 0 (0%) | 0 (0%) | 0 (0%) | 0 (0%) |
| 36+ months | 1 | 0 (0%) | 0 (0%) | 0 (0%) | 0 (0%) | 1 (100%) |
| Not reported | 2 | 1 (50%) | 0 (0%) | 0 (0%) | 0 (0%) | 1 (50%) |
| **Number of learning sessions** | | | | | | |
| 2 | 2 | 0 (0%) | 0 (0%) | 0 (0%) | 1 (50%) | 1 (50%) |
| 3 | 2 | 0 (0%) | 0 (0%) | 0 (0%) | 0 (0%) | 2 (100%) |
| 4 | 0 | 0 (0%) | 0 (0%) | 0 (0%) | 0 (0%) | 0 (0%) |
| 5 | 1 | 1 (100%) | 0 (0%) | 0 (0%) | 0 (0%) | 0 (0%) |
| Not reported | 2 | 0 (0%) | 0 (0%) | 0 (0%) | 0 (0%) | 2 (100%) |
| **Length of learning sessions** | | | | | | |
| 1/2 day | 0 | 0 (0%) | 0 (0%) | 0 (0%) | 0 (0%) | 0 (0%) |
| 1 day | 0 | 0 (0%) | 0 (0%) | 0 (0%) | 0 (0%) | 0 (0%) |
| 1-2 days | 0 | 0 (0%) | 0 (0%) | 0 (0%) | 0 (0%) | 0 (0%) |
| Not reported | 7 | 1 (14% | 0 (0%) | 0 (0%) | 1 (14%) | 5 (71%) |
| **Quality assessment** | | | | | | |
| low | 0 | 0 (0%) | 0 (0%) | 0 (0%) | 0 (0%) | 0 (0%) |
| medium | 3 | 1 (33%) | 0 (0%) | 0 (0%) | 1 (33%) | 1 (33%) |
| high | 4 | 0 (0%) | 0 (0%) | 0 (0%) | 0 (0%) | 4 (100%) |
